# Diet Control More Intensively Disturbs Gut Microbiota Than Genetic Background in Wild Type and ob/ob Mice

**DOI:** 10.3389/fmicb.2019.01292

**Published:** 2019-06-07

**Authors:** Jing-Hua Wang, Na Rae Shin, Soo-Kyoung Lim, Ungjin Im, Eun-Ji Song, Young-Do Nam, Hojun Kim

**Affiliations:** ^1^Department of Rehabilitation Medicine of Korean Medicine, Dongguk University, Goyang-si, South Korea; ^2^Department of Research and Development, Cure Pharmtech, Goyang-si, South Korea; ^3^Department of East-West Medical Science, Graduate School of East-West Medical Science, Kyung Hee University, Seoul, South Korea; ^4^Research Group of Healthcare, Korea Food Research Institute (KFRI), Seongnam-si, South Korea

**Keywords:** gut microbiota, high fat diet, high sucrose diet, overweight, sequencing

## Abstract

Changes in environmental and genetic factors are vital to development of obesity and its complications. Induction of obesity and type 2 diabetes by both leptin deficiency (ob/ob) and high fat diet (HFD) has been verified in animal models. In the present experiment, three types of diets (normal diet; ND, HFD and high sucrose diet; HSD) and two types of genetic mice (Wild type: WT and ob/ob) were used to explore the relationship among diet supplements, gut microbiota, host genetics and metabolic status. HFD increased the body, fat and liver weight of both ob/ob and WT mice, but HSD did not. HFD also resulted in dyslipidemia, as well as increased serum transaminases and fasting glucose in ob/ob mice but not in WT mice, while HSD did not. Moreover, HFD led to brain BDNF elevation in WT mice and reduction in ob/ob mice, whereas HSD did not. Both HFD and HSD had a greater influence on gut microbiota than host genotypes. In detail, both of HFD and HSD alteration elucidated the majority (≥63%) of the whole structural variation in gut microbiota, however, host genetic mutation accounted for the minority (≤11%). Overall, diets more intensively disturbed the structure of gut microbiota in excess of genetic change, particularly under leptin deficient conditions. Different responses of host genotypes may contribute to the development of metabolic disorder phenotypes linked with gut microbiota alterations.

## Introduction

Obesity and its related complications, such as type 2 diabetes and hyperlipidemia, have become an issue worldwide and the associated morbidity rate has been increasing rapidly in recent decades (Afshin et al., 2017; Zheng et al., 2018). Although the fundamental reasons for being overweight and obese are disruptions of energy balance, many etiological factors, such as genes, metabolism, the environment and dietary habits directly or indirectly lead to overweight and obesity (Brantley et al., 2005; Hill et al., 2012; Weinsier et al., 2012). Leptin is a representative energy expenditure hormone that is primarily secreted by adipocytes (Pandit et al., 2017). Because of its appetite suppressing effect, leptin deficient obese mice (ob/ob) have been deemed an excellent mutant model and utilized extensively in studies of obesity and diabetes (Drel et al., 2006). Moreover, immoderate ingestion of high fat (HFD) or HFD together with high sucrose diet (HSD) results in dietary obesity and type 2 diabetes (Xi et al., 2004; Yang et al., 2012). Interestingly, ingestion of HSD alone without over ingestion of calories also causes glucose intolerance (Sakamoto et al., 2012); however, it is still unclear if obesity can be induced by HSD alone rather than excess calories.

Over 100 trillion microbes live in the gastrointestinal tract in a symbiotic relationship with the host, and these organisms, like Firmicutes, Bacteroidetes, and Actinobacteria, etc., have been shown to play vital roles in physiological and pathological process, such as immunomodulation and maintenance of homeostasis in both animal and human subjects (Ley et al., 2006; Thursby and Juge, 2017; Park, 2018). The development of systemic metabolic disorders such as obesity and type 2 diabetes has been shown to be closely linked with changes in gut microbiota as well, representatively Firmicutes and Bacteroidetes ratio involved in obesity (Cani et al., 2007b; Parks et al., 2013; Tai et al., 2015). For example, gut microbiota reduce leptin sensitivity and the fat-suppressing neuropeptides proglucagon and brain-derived neurotrophic factor (BDNF) (Schele et al., 2013). Therefore, gut microbiota have been deemed a non-negligible factor that can contribute to obesity and its complications.

Several previous preclinical studies have shown that gut microbiota is changed in obese individuals in response to deficiencies in leptin and over consumption of HFD or HFD together with HSD (Boulange et al., 2016; Collins et al., 2016; Guo et al., 2017). In detail, host leptin deficiency changes the gut microbiota correlated with variation in long-term glucose levels, glucose intolerance and mucosal regulatory immunity and HFD plus HSD treatment more rapidly alter gut microbiota together with muscle integrity, inflammation even 3 days. As we know, genetic and environmental factors have been shown to be essential to the development of chronic metabolic disorders in numerous studies (Maes et al., 1997; Speakman, 2004). Moreover, the gut microbiota have been shown to be shaped by both genetic and dietary factors (Goodrich et al., 2014; Graf et al., 2015). Development of obesity and a tight relationship with gut microbiota alteration by both leptin deficiency (ob/ob) and HFD/HFD+HSD has also been verified (Park et al., 2016; Lu et al., 2017). However, it is not clear if gut microbiota can be altered by HSD alone when coupled with ingestion of normal calories. Hypothetically, consumption of HSD within a normal calorie range should not induce fat accumulation; therefore, it is worth investigating whether such increased accumulation occurs as a result of alterations in gut microbiota.

A previous study revealed that HFD alone induced changes in gut microbiota relevant to metabolic syndrome phenotype development, and that these changes were more important than host gene mutations in ApoA-I knockout mice (Zhang et al., 2010). In addition, gut microbiota-associated bile acid deconjugation accelerates fat synthesis in normal diet fed ob/ob mice (Park et al., 2016). However, no studies have been conducted to evaluate the effects of different diets on host-gut microbiota interactions in ob/ob mice and their wild-type (WT) lean control mice as they relate to the development of obesity. Consequently, in this study, we investigated the importance of host gene and diet on gut microbiota as it relates to obesity and other metabolic factors in ob/ob and wild type mice. Moreover, HFD and HSD were employed as different diet controls that provided different levels of calories to evaluate the contributions of diet perturbed gut microbiota and host gene mutations relevant to the development of obesity.

## Materials and Methods

### Animals and Experimental Schedule

**FIGURE 1:**
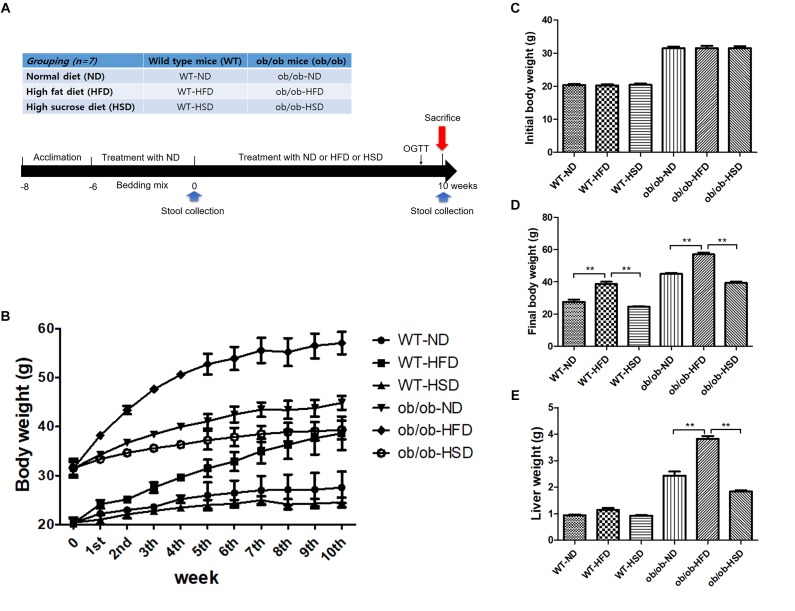
Experimental design, body and liver mass. Grouping of mice and treatment schedule **(A)**. Body weights of mice were recorded from the beginning of the experiment to week 10 **(B)**, initial week **(C)**, and final week **(D)**. Liver weights of mice were recorded from the final week **(E)**. Data were expressed as the means ± SEM and statistically evaluated using one-way ANOVA followed by Bonferroni’s *post hoc* test. ^∗∗^*P* < 0.01 (*n* = 7).

Twenty-one male C57BL/6J mice (4-weeks old, wild type) and 21 C57BL/6J-ob/ob mice (4 weeks old, genetically obese with leptin-deficiency) were obtained from the Korea Research Institute of Bioscience and Biotechnology (Ochang-eup, South Korea). The normal diet (AIN-93G, 16 cal% as fat; 20 cal% as protein, 64 cal % as carbohydrate, 4000 Kcal/kg), HFD (60 cal% as fat; 20 cal% as protein, 20 cal % as carbohydrate, 5333 Kcal/kg), and HSD (12 cal% as fat; 19 cal% as protein, 70 cal % as carbohydrate, 3702 Kcal/kg) were purchased from Todobio (Supplementary Table S1, Guri-si, South Korea). Seven animals with same genetic background in each group were housed in the same cage. To reduce individual variations in gut microbiota, the bedding of animals was thoroughly mixed twice per week for 6 weeks within the same genetic type until the start of experiment. After 6 weeks of acclimation at 22 ± 2°C under a 12-h light/12-h dark cycle and 40–60% relative humidity with free access to water and normal diet, the wildtype and ob/ob mice were divided into a normal, HFD and HSD group that received a normal diet (AIN-93G), an HFD diet and an HSD diet, respectively. All of the diets were provided *ad libitum* for 10 weeks (Figure 1A).

The body weight and food intake were recorded once a week for 10 weeks. On the last experimental day, the animals were sacrificed under the anesthetics Zoletil (tiletamine-zolazepam, Virbac, Carros, France) and Rompun (xylazine-hydrochloride, Bayer, Leverkusen, Germany) (1:1, v/v). Blood was collected from the abdominal aorta and rapidly transferred into a BD Vacutainer (Franklin Lakes, NJ, United States), after which the brains and fats were removed and quickly stored in liquid nitrogen. The livers were then removed and weighed. After 2 h of clotting, sera were separated from whole blood by centrifugation at 3000 ×*g* for 15 min. The Food Efficiency Ratio (FER) was computed by dividing the average body weight gain by the average food intake for each group.

The animal experimental protocol was approved by the Institutional Animal Ethical Committee of Dongguk University (Approval No. IACUC-2014-030). In addition, all experiments were conducted according to the Guide for the Care and Use of Laboratory Animals (Institute of Laboratory Animal Resources, Commission on Life Sciences, National Research Council, United States; National Academy Press: Washington, DC, 1996).

### Serum Biochemical Analysis

Blood was collected from ventral aorta under Zoletil and Rompun anesthesia. After 1 h of clotting at room temperature, blood was centrifuged at 3000 ×*g* for 15 min for serum separation. The serum levels of triglyceride (TG), total cholesterol (TC), high density lipoprotein (HDL), low density lipoprotein (LDL), aspartate transaminase (AST), and alanine transaminase (ALT) were determined using commercial enzymatic assay kits according to the manufacturer’s instructions (Asan Pharmaceutical Co., Seoul, South Korea).

### Oral Glucose Tolerance Test (OGTT)

Mice were orally dosed with glucose solution (2 g/kg, Sigma, United States) before 12 h of fasting in the start of the last week. The blood glucose levels in tail blood drops were then measured using an ACCU-CHEK Active blood glucose meter (Mannheim, Germany) at 0, 30, 60, 90, and 120 min after administration. The OGTT results were also expressed as areas under the curves (AUC) to estimate the extent of the glucose tolerance impairment.

### Western Blot

Mouse brain cortexes were homogenized in RIPA buffer containing protease and phosphatase inhibitor. The supernatants were subsequently isolated, after which total protein concentrations were measured using a BCA kit (Thermo Scientific, Rockford, LL, United States). Denatured proteins were then separated in 10% SDS-PAGE gel, after which they were transferred to a polyvinylidene fluoride (PVDF) membrane (GE Healthcare Life Science, Germany) using a Mini-PROTEAN Tetra Cell system (BioRad Laboratories Inc., Hercules, CA, United States). Next, membranes were blocked with 5% skim milk containing TBST, Tris–buffered saline and Tween 20 for 1 h, after which they were treated overnight with primary antibody (anti-BDNF) at 4°C (1:200; Santa Cruz Biotechnology, Inc., Santa Cruz, CA, United States) and anti-β-actin (1:1,000; Santa Cruz Biotechnology, Inc.), then incubated with anti-rabbit IgG-peroxidase conjugated secondary antibody for 1 h (1:2,000; Santa Cruz Biotechnology, Inc.). Finally, the membranes were detected using SUPEX ECL solution and photographed with a LAS3000 Imager (FUJIFILM, FUJI, Japan).

### Metagenomic Analysis of Gut Microbiota and Functional Predictive Annotation

Fecal samples were collected and kept at -80°C before DNA extraction. The genomic DNA was then isolated using a QIAamp stool DNA mini kit (QIAGEN, Hilden, Germany) according to the manufacturer’s instructions. The V1–V2 region of the 16S rRNA genes was amplified using a Thermal Cycler PCR system (BioRad, Hercules, CA, United States), after which amplicons were purified using a LaboPass PCR purification kit (COSMO GENTECH, Seoul, South Korea). An equimolar concentration of each amplicon from different samples was pooled to equal proportions based on their molecular weight and purified using Agencourt AMPure XP PCR purification beads (Agencourt Bioscience, Beverly, MA, United States). The DNA concentration and quality were confirmed on a BioAnalyzer 2100 microfluidics device (Agilent, Santa Clara, CA, United States) using a DNA 100 lab chip (Agilent, Santa Clara, CA, United States). The mixed amplicons were amplified on sequencing beads by emulsion PCR (emPCR). Sequencing reactions were performed using an Ion Torrent Next-Generation Sequencing Platform (Ion PGM, Life Technologies, Carlsbad, CA, United States).

Operational taxonomic units selection, taxonomic assignment and phylogenetic reconstruction were conducted using the QIIME1 (Version 1.9.1, University of Colorado, Boulder, CO, United States) software package and visualized with the LEfSe (linear discriminant analysis effect size) program (Hutlab, Boston, MA, United States), STAMP v2.1.3 software (Dalhousie University, Halifax, Canada) and Graphpad Prism 5. All metagenomics data were predictably profiled with PICRUSt-1.0.0 (Phylogenetic Investigation of Communities by Reconstruction of Unobserved States) (Langille et al., 2013) and HUMAnN2 (The HMP Unified Metabolic Analysis Network 2) (Abubucker et al., 2012). The functional and taxonomic differences in predicted results were statistically analyzed and represented graphically using the STAMP v2.1.3 software (Dalhousie University, Halifax, Canada^1^).

All sequencing raw reads have been deposited in the European Nucleotide Archive (ENA) under the project accession number PRJEB28486 with a unique name (ena-STUDY-DGU-05-09-2018-03:21:13:852-167).

### Statistical Analysis

All animal experimental data were analyzed by one-way ANOVA followed by Bonferroni’s *post hoc* test using GraphPad Prism version 5.01 (La Jolla, CA, United States). The results were expressed as the means ± standard error of the mean (SEM), and a *P* < 0.05 and *P* < 0.01 was considered statistically significant. Relationship strength between parameters was evaluated by the two tailed Pearson’s correlation test. An absolute value of Pearson’s correlation coefficient *R* > 0.4 was considered to indicate a positive correlation with connected by red line while *R* < -0.4 was considered a negative correlation with connected by blue line. The boldness of the line indicates the strength of the correlation.

## Results

### HFD, but Not HSD, Changed Body and Liver Weight in Both ob/ob and WT Mice

All ob/ob mice and their WT lean control mice were fed either ND, HFD, or HSD (*n* = 7 for each group) for 10 weeks. Each WT or ob/ob mouse had a similar body weight at week 6; however, the body weight of mice in the HFD groups was significantly increased by HFD relative to the ND groups after 10 weeks, regardless of genetic type, but not by HSD (Figure 1B–D). Additionally, feeding ob/ob mice HFD resulted in markedly elevated liver weight relative to the corresponding normal group, but no significant augmentation of liver weight was observed in any WT groups (Figure 1E).

### HFD, but Not HSD, Elevated Caloric Intake and FER in Both ob/ob and WT Mice, but Did Not Change Food Intake by WT Mice

**FIGURE 2:**
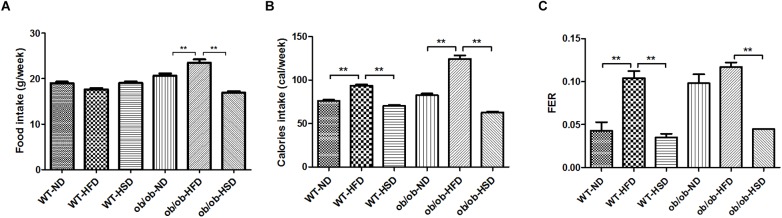
Food and calorie intake and food efficiency ratio. Food intakes were recorded twice per week **(A)**, the calorie intakes were calculated according to the percentage of calories in each diet **(B)**, and the food efficiency ratio (FER) was calculated by dividing body weight gain (g/day) by food intake (g/day) **(C)**. Data were expressed as the means ± SEM and statistically evaluated using one-way ANOVA followed by Bonferroni’s *post hoc* test. ^∗∗^*P* < 0.01 (*n* = 7).

Although noticeable differences in absolute volume of food intake were not found in WT groups, absolute volume of food intake was markedly increased by HFD relative to ND and HSD in ob/ob groups (Figure 2A). Additionally, treatment with HFD resulted in notably increased caloric intake and FER relative to other diets in both WT and ob/ob mice (Figure 2B,C).

### HFD, but Not HSD, Increased the Fat Weights in Both ob/ob and WT Mice

**FIGURE 3:**
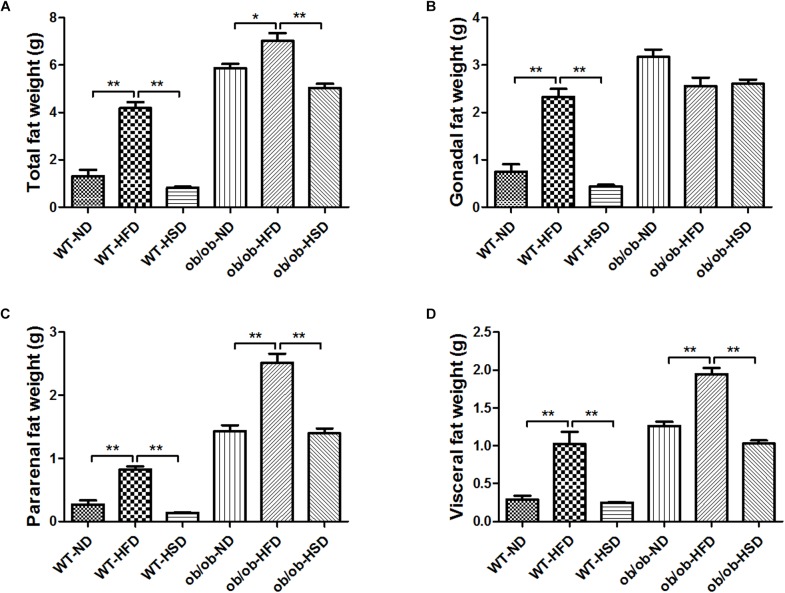
Adipose tissues weights. At the end of the experiment, total fat weight **(A)**, gonadal fat **(B)**, pararenal fat **(C)**, and visceral fat **(D)** mass were determined and calculated. Data were expressed as the means ± SEM and statistically evaluated using one-way ANOVA followed by Bonferroni’s *post hoc* test. ^∗^*P* < 0.05; ^∗∗^*P* < 0.01 (*n* = 7).

Providing WT and ob/ob mice with HFD resulted in increased total fat weight, pararenal fat weight and visceral fat weight relative to other diet groups (Figure 3A–D). Although HFD treatment led to marked increases in gonadal fat weight relative to other diet groups in WT mice, no significant enhancement of gonadal fat weight was found in ob/ob mice (Figure 3A–D).

### HFD, but Not HSD, Led to Dyslipidemia in ob/ob Mice, but Not in WT Mice

**FIGURE 4:**
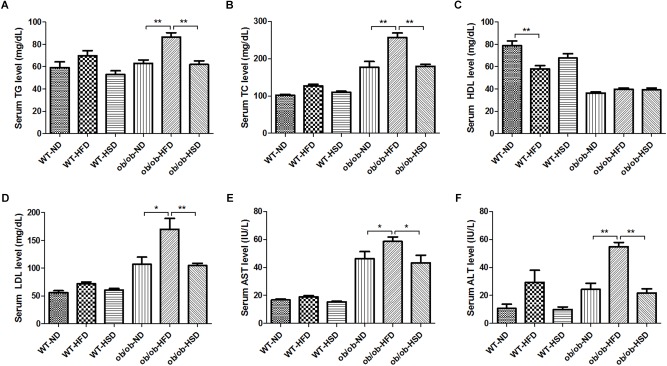
Serum biochemistry parameters. At the end of the experiment, blood was collected from the ventral aorta under Zoletil and Rompun anesthesia. After separation of serum, the serum level of triglyceride (TG) **(A)**, total cholesterol (TC) **(B)**, high density lipoprotein (HDL) **(C)**, low density lipoprotein (LDL) **(D)**, aspartate transaminase (AST) **(E)**, and alanine transaminase (ALT) **(F)** were determined using commercial enzymatic assay kits. Data were expressed as the means ± SEM and statistically evaluated using one-way ANOVA followed by Bonferroni’s *post hoc* test. ^∗^*P* < 0.05; ^∗∗^*P* < 0.01 (*n* = 7).

Feeding ob/ob mice HFD resulted in a marked increase in the level of serum TG, TC, LDL, AST, and ALT relative to normal diet, but no such changes were observed in response to HSD (Figure 4A–F). Moreover, feeding WT mice HFD led to slight elevation of serum TG, TC, LDL, AST, and ALT levels, but these changes were not statistically significant (Figure 4A–F). In contrast, feeding WT mice HFD notably reduced the serum HDL level relative to the corresponding normal diet, but these changes were not observed in HSD. However, feeding ob/ob mice different types of diet did not induce a significant difference in serum HDL levels.

### HFD, but Not HSD, Resulted in Elevated Fasting Glucose Levels in ob/ob Mice and OGTT (AUC) in WT Mice

**FIGURE 5:**
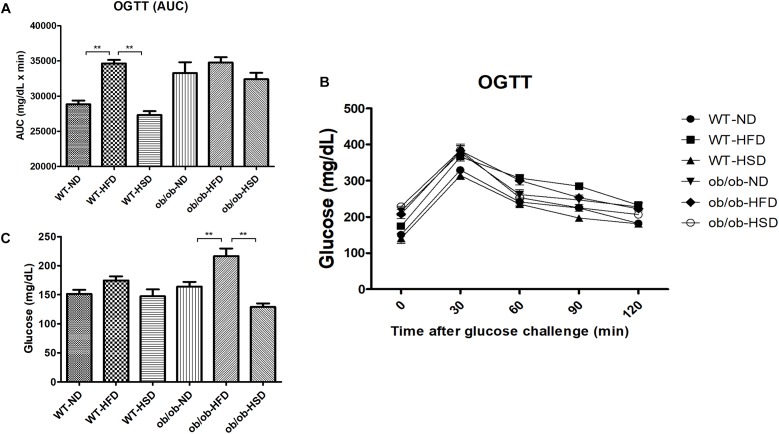
Fasting glucose and OGTT. Fasting blood glucose **(C)** was determined on the last day of experiment. Oral glucose tolerance tests (OGTTs) **(A)** were performed on the animals during the last week and the areas under the curves (AUCs) **(B)** were constructed as described in the see section “Materials and Methods”. Data were expressed as the means ± SEM and statistically evaluated using one-way ANOVA followed by Bonferroni’s *post hoc* test. ^∗∗^*P* < 0.01 (*n* = 7).

Feeding ob/ob mice a HFD resulted in significant elevation of the fasting blood glucose level when compared to the corresponding normal diet, but HSD did not (Figure 5A). However, in WT mice groups, HFD only weakly increased the level of fasting blood glucose relative to other diet groups, but this increase was not significant. In addition, impairment of the AUC of OGTT was calculated to compare the extent of glucose tolerance (Figure 5B,C). Feeding WT mice HFD resulted in a significant increase in AUC when compared to other diet groups (Figure 5C). In ob/ob mice, all groups showed a high level of OGTT (AUC) relative to WT groups; however, no significant difference in OGTT (AUC) was detected among these ob/ob groups (Figure 5C).

### HFD, but Not HSD, Resulted in Brain BDNF Elevation in WT Mice and Reduction in ob/ob Mice

**FIGURE 6:**
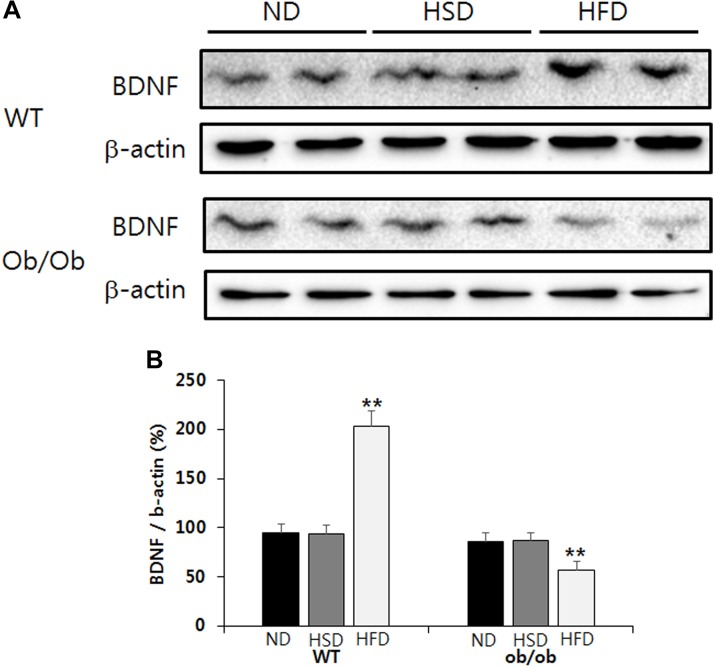
Western blot. The relative BDNF content in the brain cortex was analyzed by western blot **(A)**. The relative density of protein bands was calculated using the Quantity One 1-D Analysis Software and normalized with β-actin **(B)**. Data were expressed as mean ± SEM and statistically evaluated using one-way ANOVA followed by Bonferroni’s *post hoc* test. ^∗∗^*P* < 0.01 compared to ND and HSD (*n* = 7).

Feeding WT mice HFD resulted in a significant increase of BDNF content in the brain cortex relative to the corresponding normal diet (Figure 6A,B). Conversely, feeding ob/ob mice HFD resulted in a significant decrease of BDNF content in the brain cortex when compared to the corresponding normal diet (Figure 6A,B). However, the level of BDNF in the brain cortex was not altered in either WT or ob/ob mice treated with HSD (Figure 6A,B).

### Whole Structural Responses of Gut Microbiota to Intake of Various Diets and Host Leptin Deficiency

**FIGURE 7:**
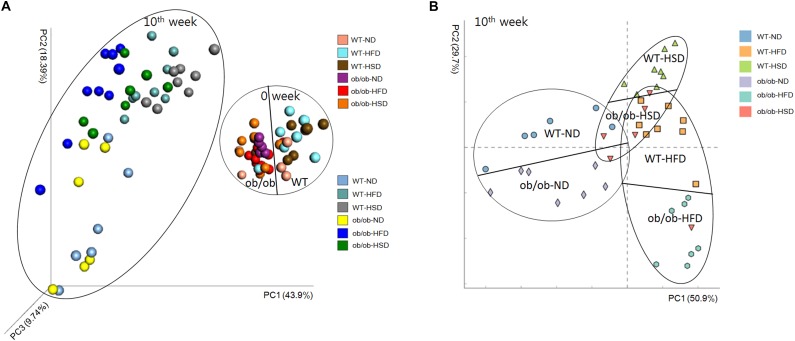
Weighted Unifrac Principal coordinate analysis chart. At the start and end of the experiment, mice fecal samples were collected and the microbial communities were analyzed by 16S rRNA gene sequencing as described in the see section “Materials and Methods”. **(A)** Both week 0 and week 10 and **(B)** only week 10 data were analyzed by the Weighted Unifrac PCoA method and plotted using XLSTAT to further evaluate the similarity between bacterial communities.

Principal coordinate analysis based on 16S rRNA gene sequence data revealed obvious differences in the composition of gut microbiota among animal types, food types and time points. Comparison of samples collected on the initial and final day of experiment revealed time-associated differences (Figure 7 and Supplementary Figure S2). Before starting the experiment, non-obvious differences were found among all groups, regardless of whether mice were ob/ob or WT. However, at the end of the experiment, HFD-related differences were mainly found along PC1, which accounted for 63.2% of the total variations, whereas the two genotypes showed an obviously smaller difference along PC3, which accounted for only 10.6% of the total variations (Supplementary Table S2). Moreover, HSD-related differences were mainly observed along PC1, which accounted for 66.4% of the total variations, whereas the two genotypes showed smaller differences along PC3, which accounted for only 5.4% of the variations (Supplementary Table S2).

**FIGURE 8:**
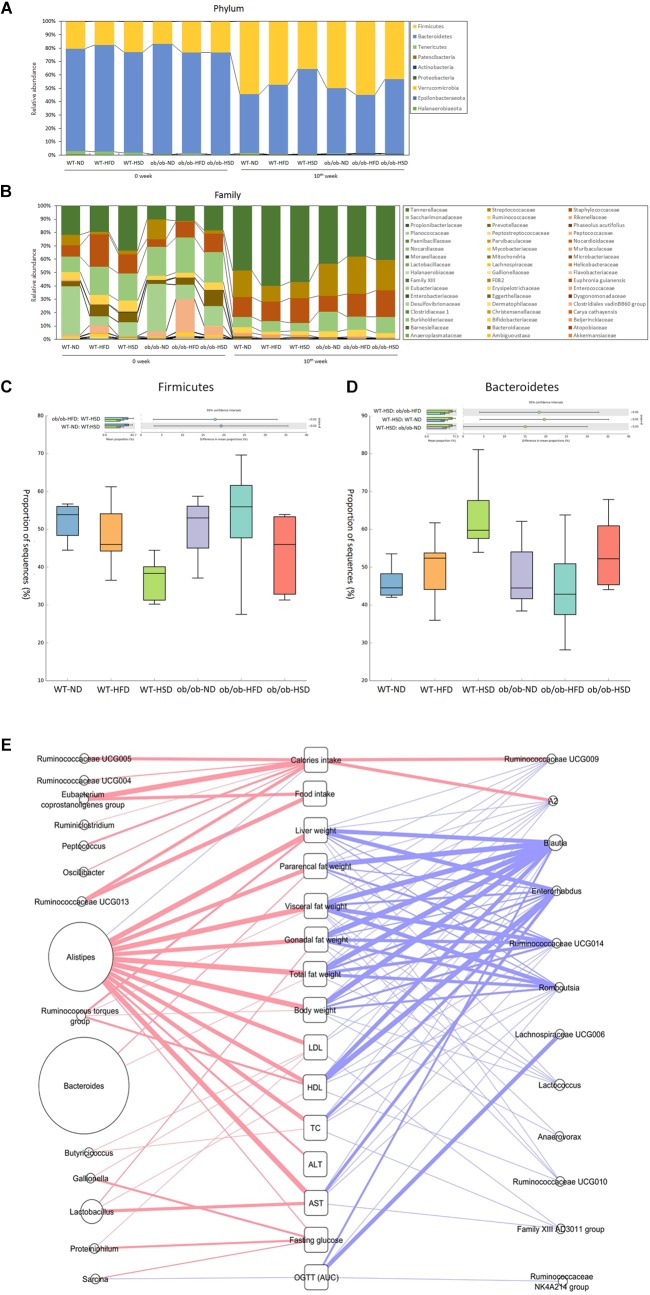
Phylum and family level composition of fecal microbiota and Pearson’s correlation analysis. Gut microbiota taxonomic profiles. The relative abundance (%) of fecal bacterial phyla **(A)** and families **(B)** were compared according to the 16S rRNA gene sequencing data. The proportion of sequences (%) of firmicutes **(C)** and Bacteroidetes **(D)** were compared. Taxonomic differences in results were statistically analyzed and represented graphically using the STAMP v2.1.3 software (Dalhousie University, Halifax, Canada). **(E)** All of the gut microbiota composition and host parameters data were evaluated using Pearson’s correlation analysis, with a *R* value greater than 0.4 indicating a positive correlation (red line) and a *R* value less than –0.4 indicating a negative correlation (blue line). The boldness of the line indicates the strength of the correlation. The size of each circle indicates the average relative abundance of each genus.

At the phylum-level, feeding WT mice with HSD resulted in a significant decrease in Firmicutes and an increase in Bacteroidetes relative to the corresponding normal diet, but HFD did not. However, feeding ob/ob mice with HSD generated a similar pattern as WT mice, but this difference was not statistically significant (Figure 8). At the low taxonomic-level, ob/ob mice showed a significantly higher level of *Alistipes* and a lower level of *Blautia* in the two ND groups, while ob/ob mice showed a significantly higher level of *Lactobacillus* and lower level of *Lachnospiraceae* and *Muribaculaceae* in the two HFD groups (Figure 8). However, no significant differences in genera were found between WT and ob/ob mice in the HSD groups. Thus, it is supposed that HSD more sensitively regulated the phylum-level of gut microbiota in WT mice than ob/ob mice. Although the mechanism is still not clear, it is shown that leptin gene co-effect with HSD play a critical role in changing gut microbiota in phylum-level. However, genus-level of gut microbiota was not obviously influenced by HSD together with leptin gene expression or not. Moreover, various alpha diversity indexes revealed no notable differences in response to any diets, genotypes or time points (Supplementary Figure S1). Taken together, HSD co-effect with leptin only changed gut microbiota in high taxonomic level. Nevertheless diets, genotypes didn’t evidently change the gut microbial community richness, evenness and diversity even in different time points in the scale of entire ecosystem.

In addition, according to the result of Pearson’s correlation analysis between gut microbiota composition and host metabolic parameters, Alistipes showed a evident positive correlation (Pearson *r* > 0.4) with extensive weights and serum biochemical parameters rather than other gut bacteria, inversely Blautia and Enterorhabdus showed a distinct negative correlation (Pearson *r* < 0.4) (Figure 8E). However, relative high percentage of Bacteroides only positively correlated with fat weight, especially pararenal fat weight (Figure 8E).

## Discussion

Epidemiological data for the past 30 years reveal that obesity, a popular chronic metabolic disease according to the American Medical Association (Stoner and Cornwall, 2014), is becoming an increasing problem in the worldwide in developed and developing countries (Ng et al., 2014). In the last decade, many investigators have focused on gut microbiota and chronic metabolic diseases, including obesity and type 2 diabetes, in an attempt to identify complex mechanisms that can be targeted to treat the condition (Baothman et al., 2016).

Turnbaugh et al. (2006) showed that changes in the ratio of Firmicutes and Bacteroidetes contributed to obesity; specifically, notable elevation of Firmicutes and reduction of Bacteroidetes were observed in obese subjects relative to lean subjects, while HFD also promoted an increase in Firmicutes and reduction of Bacteroidetes and the changes in the dominant phyla promoted more effective caloric intake, leading to increased weight and obesity (Turnbaugh et al., 2006; Tilg and Kaser, 2011). However, Zhang et al. found no phylum-wide gut microbiota alteration in HFD induced obese animals, regardless of genotypes (Apoa-I^-/-^) (Zhang et al., 2010). Interestingly, we also did not observe significant changes in Firmicutes and Bacteroidetes in HFD-induced obese animals, regardless of genotypes (ob/ob^-/-^). Nevertheless, our study revealed that feeding both WT and ob/ob mice HSD decreased Firmicutes and increased Bacteroidetes relative to ND, but this genotype (ob/ob^-/-^) showed a different degree of phylum-wide gut microbiota change when compared to the WT. A previous study showed the gut microbiota structure could be significantly changed by variations in caloric intake (Jumpertz et al., 2011), while another study reported that calorie restriction can modulate the balance of gut microbiota in a way that exerts health benefits to the host (Zhang et al., 2013). Therefore, in the present study, the dissimilar caloric intake between the HFD and HSD group might have been a vital factor influencing gut microbiota alterations. The results of the present study also suggest that reducing the caloric intake will be beneficial to maintenance of a healthy structure of gut microbiota and conducive to amelioration of obesity. Functional predictions from PICRUSt based on the 16S rRNA bacteria gene sequences indicated that HFD more widely regulated gene function than HSD, regardless of genotypes (Supplementary Figure S3). Moreover, HFD contributed more to the difference in gene function between WT and ob/ob mice than ND and HSD. Additionally, KEGG pathway predictions from HUMAnN2 showed the same pattern as the PICRUSt results (Supplementary Figure S4). Taken together, these findings indirectly illustrated that HFD exerts more important actions on functional alterations of gut microbiota.

In fact, alpha diversity is a method to evaluate the species richness and evenness in a single group. Similar to the results of other studies (Kubeck et al., 2016), alpha-diversity analysis revealed that HFD did not cause any dissimilarity in the richness and evenness of species in each single group between 0 and 10 weeks, regardless of genetic background. HSD also showed the same pattern as HFD upon various alpha-diversity analysis. In the present study it has been demonstrated that the species richness and evenness of gut microbiota in each single group cannot be changed by feeding with HFD or HSD for at least 10 weeks, regardless of genetic difference.

Endotoxin is a complex lipopolysaccharide generated by the cell membranes of Gram-negative bacteria (Erridge et al., 2007). The role of endotoxin in the development and beginning of chronic metabolic diseases has been acknowledged (Cani et al., 2007a; Gomes et al., 2017), and it has been reported that HFD/HFD+HSD altered gut microbiota triggered inflammation and prompted low grade endotoxemia, which is a vital mechanism for accelerating development of obesity and related metabolic disorders (Cani et al., 2007a; Sanchez-Tapia et al., 2017). Our study also revealed that both HFD and HSD altered the composition of gut microbiota relative to ND. When compared to ND, HFD markedly augmented endotoxin producing bacteria belonging to Bacteroidaceae and Tannerellaceae while diminishing the non-endotoxin producing bacteria Erysipelotrichaceae, both in WT and ob/ob mice. However, HFD only significantly elevated the fasting glucose levels in ob/ob mice. Body weight and liver weight also showed a similar pattern as fasting glucose. Interestingly, differences in the caloric intake of HFD and HSD did not lead to glucose intolerance or elevation of fasting glucose, indicating that high caloric intake elevated the risk of obesity, hepatosteatosis and type 2 diabetes.

Although some studies have shown that environment dominates over host genetics in shaping gut microbiota in humans (Kubeck et al., 2016), other studies have demonstrated that host genetics influence the composition of the gut microbiome and impact host metabolism (Goodrich et al., 2014; Ussar et al., 2016). According to our PCoA results, changes in HFD explained 63.2% of the total structural variation in gut microbiota, whereas genetic mutation (ob/ob: leptin deficiency) accounted for only 10.6%. A previous study of apolipoprotein A-I gene mutation (Apoa-I) mice fed HFD indicated similar findings regarding the ability of various factors to induce gut microbiota changes. Similarly, HSD changes explained 66.4% of the total structural variation in gut microbiota, whereas genetic mutation accounted for only 5.4%. Therefore, our study demonstrated that diet exerts stronger effects than genetics, and that the impact of HSD is slightly greater than that of HFD in terms of alteration of gut microbiota structure. However, in the present study food combination was not applied for exploring the interference of gut microbiota. It will be examined whether synergistic disturbance of gut microbiota by food collocation in future. All the critical consequence can be applied in precise nutrition to improve health of human being.

Commensal microbiota have been shown to alter the level of neurotrophic factors (Diaz Heijtz et al., 2011), including BDNF. Previous studies demonstrated that BDNF exerts a potent role in cognitive abilities (Borrelli et al., 2016), emotional regulation (Bjorkholm and Monteggia, 2016) and gastrointestinal function (Wang et al., 2015). Moreover, HFD or hepatocytes and hippocampal neurons prompted elevation of BDNF mRNA expression in mice (Genzer et al., 2016). In the present study, HFD resulted in significant elevation of brain BDNF in WT mice and a notable reduction in ob/ob mice, but HSD did not. Therefore, it can be speculated that HFD more noticeably regulated BDNF than HSD through regulation of the gut microbiota; however, the ability was modulated by genotypes.

Although a previous study indicated that Apoa-I knockout mice with HFD showed less severe metabolic disorder phenotypes than WT mice with HFD (Zhang et al., 2010), our results did not show the above pattern in the level of fasting glucose between ob/ob-HFD mice and WT-HFD mice. Moreover, no difference in glucose tolerance was observed between WT-HFD mice and ob/ob-HFD mice. It is possible that different genotypes had only slight effects on gut microbiota relative to diets, and that different genotypes made different contributions to the development of metabolic disorder phenotypes.

## Conclusion

The results of our study demonstrate that diet more intensively disturbs gut microbiota than genetic change, particularly deficiency of leptin in mice. Long term feeding with HFD and HSD had different impacts on the structure of gut microbiota. Specifically, HSD feed without superfluous consumption obviously changed the structure of gut microbiota; however, obesity and its complication-related parameters were not visibly altered. Leptin deficiency changes gut microbiota in response to high-fat diet, while host genotypes show different responses to contribute to the development of metabolic disorder phenotypes, possibly linked with gut microbiota alteration. Overall, interactions between host genetics, gut microbiota and diet are closely linked to the development of obesity and its complications.

## Data Availability

The datasets generated for this study can be found in European Nucleotide Archive (ENA), ena-STUDY-DGU-05-09-2018-03:21:13:852-167.

## Ethics Statement

The animal experimental protocol was approved by the Institutional Animal Ethical Committee of Dongguk University (Approval No. IACUC-2014-030). In addition, all experiments were conducted according to the Guide for the Care and Use of Laboratory Animals (Institute of Laboratory Animal Resources, Commission on Life Sciences, National Research Council, United States; National Academy Press: Washington, DC, 1996).

## Author Contributions

HK perceived and designed the study. J-HW wrote the manuscript. S-KL performed the experiments. J-HW and NS analyzed the data. UI, Y-DN, and E-JS participated in study design and data analysis. All authors have read and approved the final manuscript.

## Conflict of Interest Statement

The authors declare that the research was conducted in the absence of any commercial or financial relationships that could be construed as a potential conflict of interest.

## Footnotes

1 http://kiwi.cs.dal.ca/Software/STAMP
