# Supplementary material for: Diet Control More Intensively Disturbs Gut Microbiota Than Genetic Background in Wild Type and ob/ob Mice
Source: Front Microbiol. 2019 Jun 7;10:1292. doi: 10.3389/fmicb.2019.01292 (PMC6568241; doi:10.3389/fmicb.2019.01292)

Supplementary Materials

# Supplementary Figures and Tables

| **Supplementary Table S1 Animal diets formulation and ingredients** | | | | | | |
| --- | --- | --- | --- | --- | --- | --- |
| Formulation | normal diet (AIN-93G) | | high fat diet | | high sucrose diet | |
|  | gm% | kcal% | gm% | kcal% | gm% | kcal% |
| Protein | 20 | 20 | 27 | 20 | 17 | 19 |
| Carbohydrate | 64 | 64 | 25 | 20 | 64 | 70 |
| Fat | 7 | 16 | 36 | 60 | 5 | 12 |
| Kcal/kg | 4,000 | | 5,333 | | 3,702 | |
|  | | | | |  |  |
| Ingredient | g | kcal | g | kcal | g | kcal |
| Casein (from milk) | 200 | 800 | 200 | 800 | 170 | 680 |
| Corn starch | 397,486 | 1,590 | 47,536 | 190 | 0 | 0 |
| Sucrose | 100 | 400 | 0 | 0 | 630 | 2,520 |
| Dextrose | 132 | 528 | 132 | 528 | 50 | 450 |
| Cellulose | 50 | 0 | 50 | 0 | 100 | 0 |
| Soybean oil | 70 | 630 | 25 | 225 | 0 | 0 |
| Lard | 0 | 0 | 245 | 2,205 | 0 | 0 |
| Mineral mixture | 35 | 0 | 35 | 0 | 35 | 0 |
| Vitamin mixture | 10 | 40 | 10 | 40 | 10 | 40 |
| TBHQ | 0.014 | 0 | 0.014 | 0 | 0 | 0 |
| L-Cystine | 3 | 12 | 3 | 12 | 3 | 12 |
| Choline bitartrate | 2.5 | 0 | 2.5 | 0 | 2 | 0 |
| Total | 1,000 | 4,000 | 750.1 | 4,000 | 1,000 | 3,702 |
| Abbreviations: TBHQ, tertiary butylhydroquinone | | | | | | |

| **Supplementary Table S2 Changes of total structural variation of gut microbiota by diets and genotypes** | | |
| --- | --- | --- |
|  | ND vs HFD |  |
| HFD changes | PC1 | 63.20% |
|  | PC2 | 19.10% |
| Genetic mutation (ob/ob) | PC3 | 10.60% |
|  |  |  |
|  | ND vs HSD |  |
| HSD changes | PC1 | 66.40% |
|  | PC2 | 19.10% |
| Genetic mutation (ob/ob) | PC3 | 5.40% |

**Supplementary Figure S1 alpha diversity index comparison.**

Fecal samples were collected at the start and end of the experiment and the microbial communities were analyzed by 16s rRNA gene sequencing as described in the Materials and methods section. (A) Phylogenetic diversity whole tree, (B) Chao1 index and Observed OTUs were calculated by QIIME 1 and diagramed using Excel 2013.


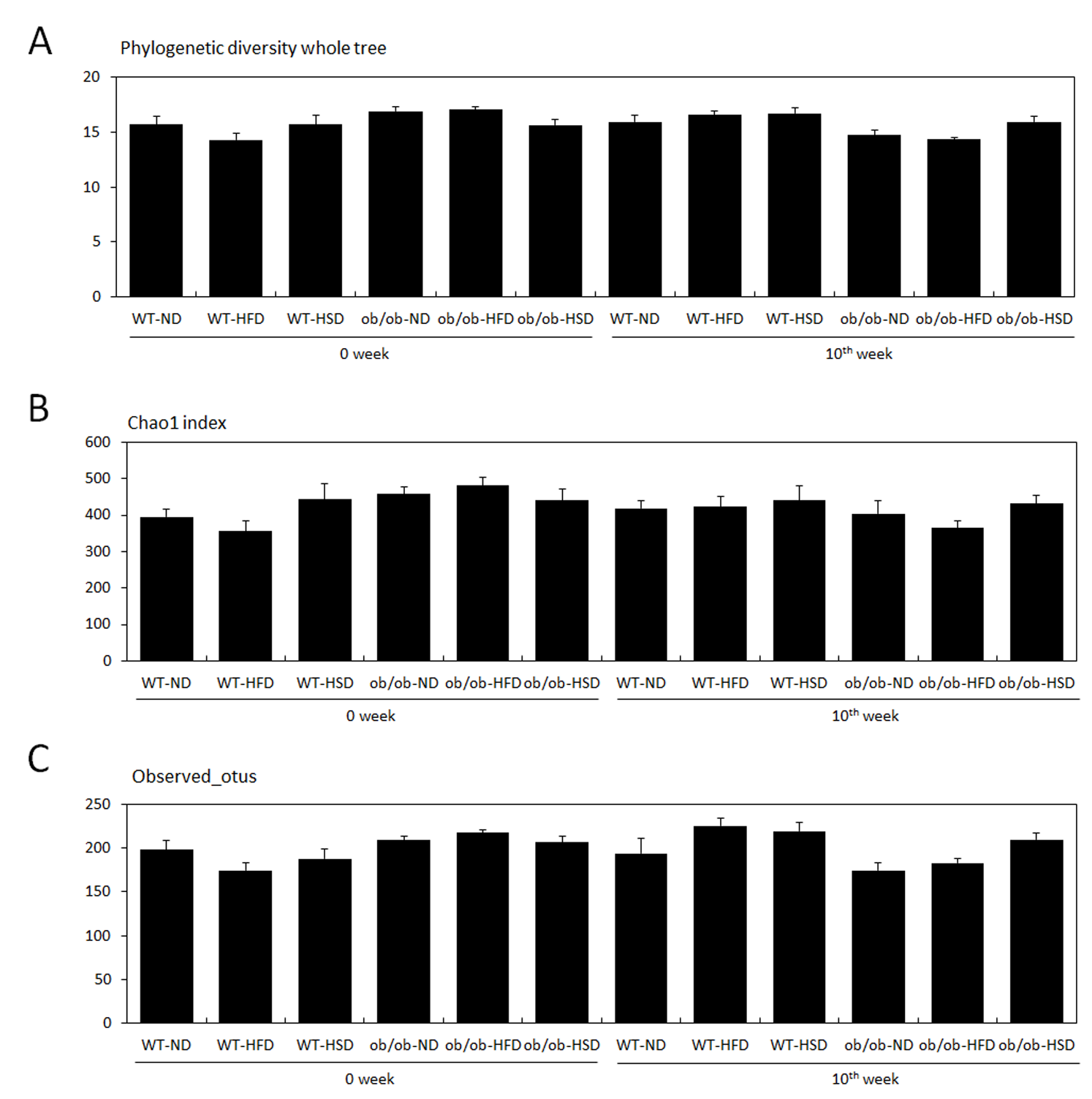


**Supplementary Figure S2 Weighted Unifrac Principal coordinate analysis.**

Start and end of experiment, the mice fecal samples were collected and the microbial communities were analyzed by 16s rRNA gene sequencing as described in the Materials and methods section. (A) ob/ob mice and (B) WT mice were separately analyzed by the Weighted Unifrac PCoA method and diagramed using XLSTAT to further evaluate the similarities between bacterial communities.


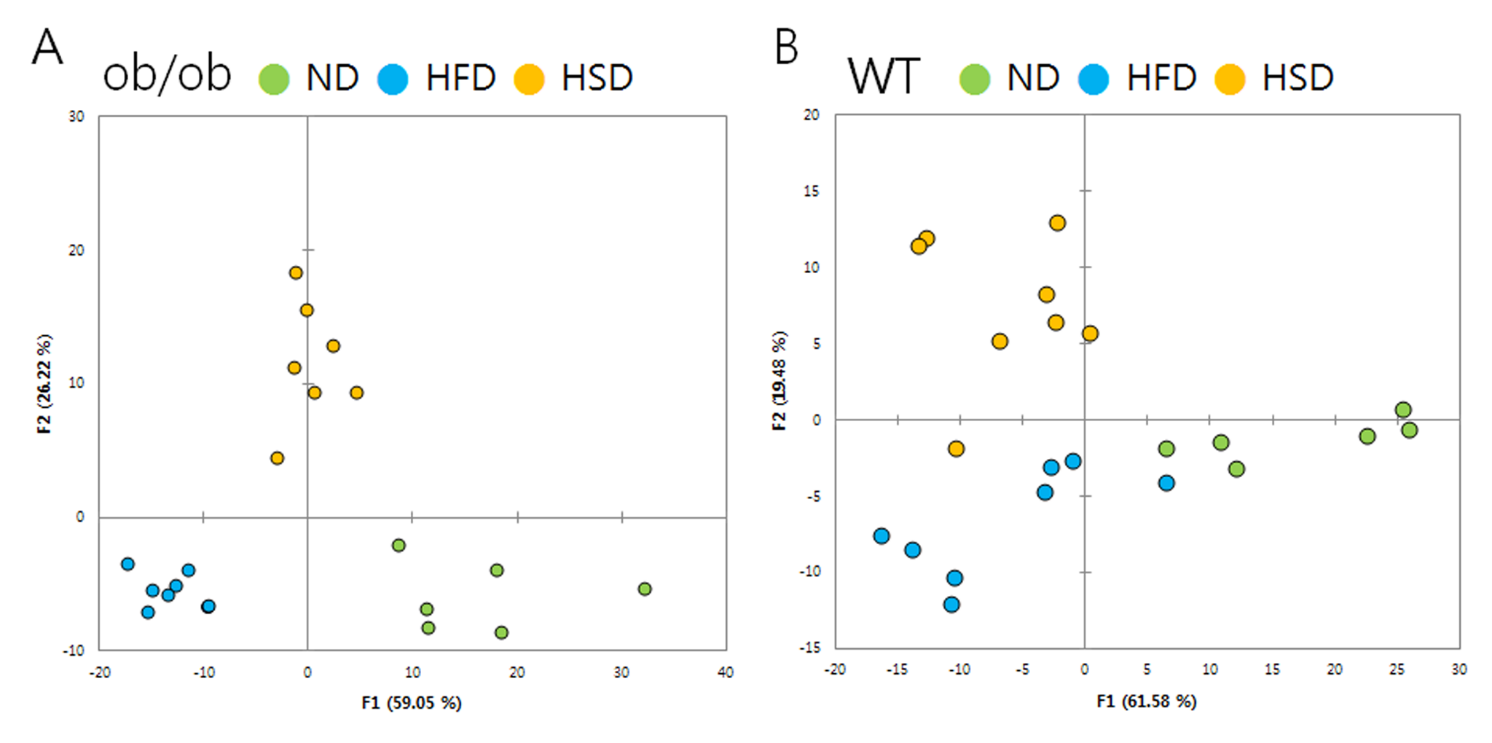


**Supplementary Figure S3 PICRUSt analysis for function prediction.**

All of the 16s rRNA gene sequencing data were predictably profiled by PICRUSt-1.0.0 (Phylogenetic Investigation of Communities by Reconstruction of Unobserved States) and statistically analyzed and represented graphically using the STAMP v2.1.3 software (Dalhousie University, Halifax, Canada). (A) WT-ND vs ob/ob-ND (B) WT-HFD vs ob/ob-HFD (C) WT-HSD vs ob/ob-HSD (D) WT-ND vs WT-HFD (E) ob/ob-ND vs ob/ob-HFD (F) WT-ND vs WT-HSD (G) ob/ob-ND vs ob/ob-HSD.


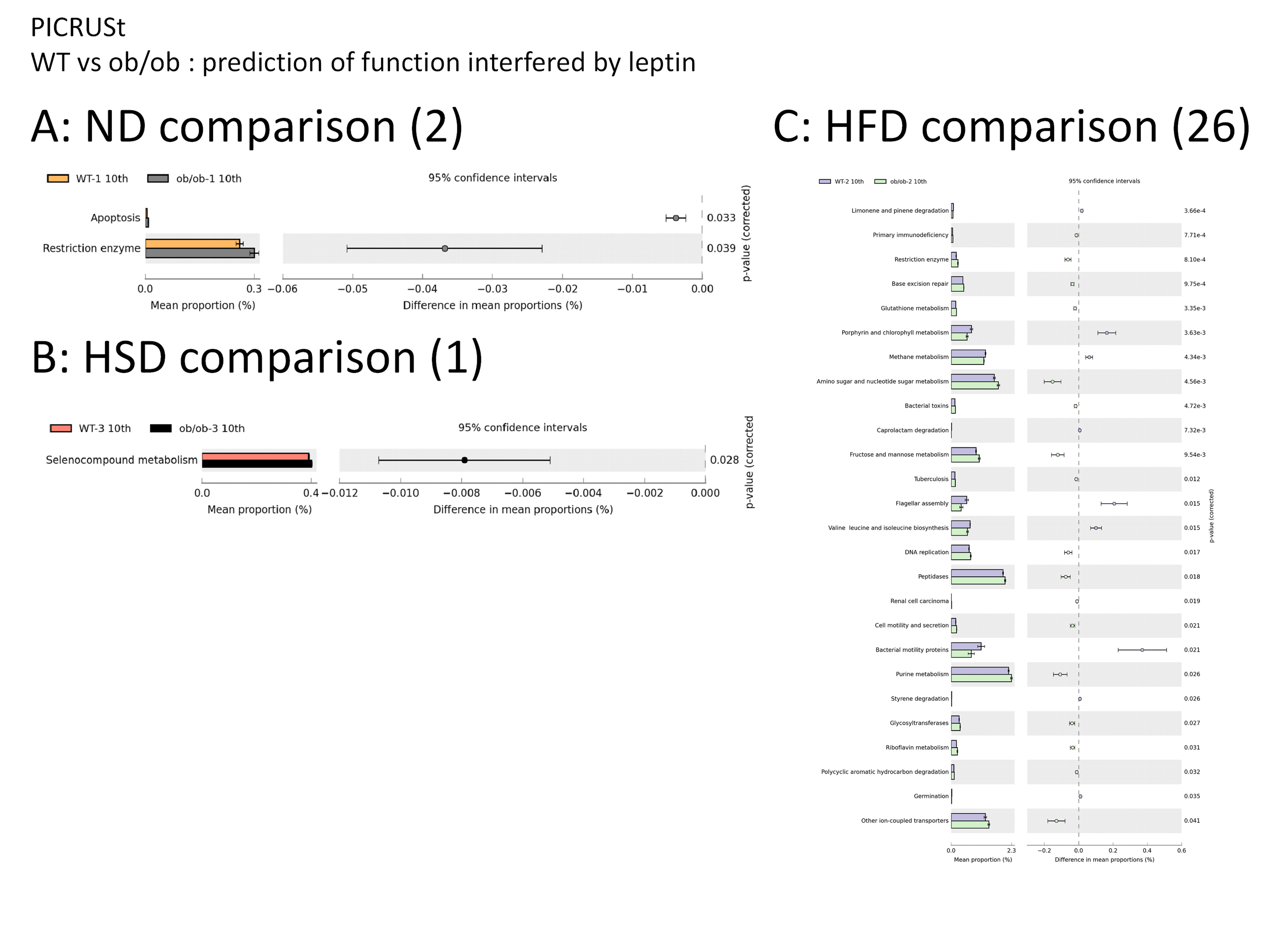


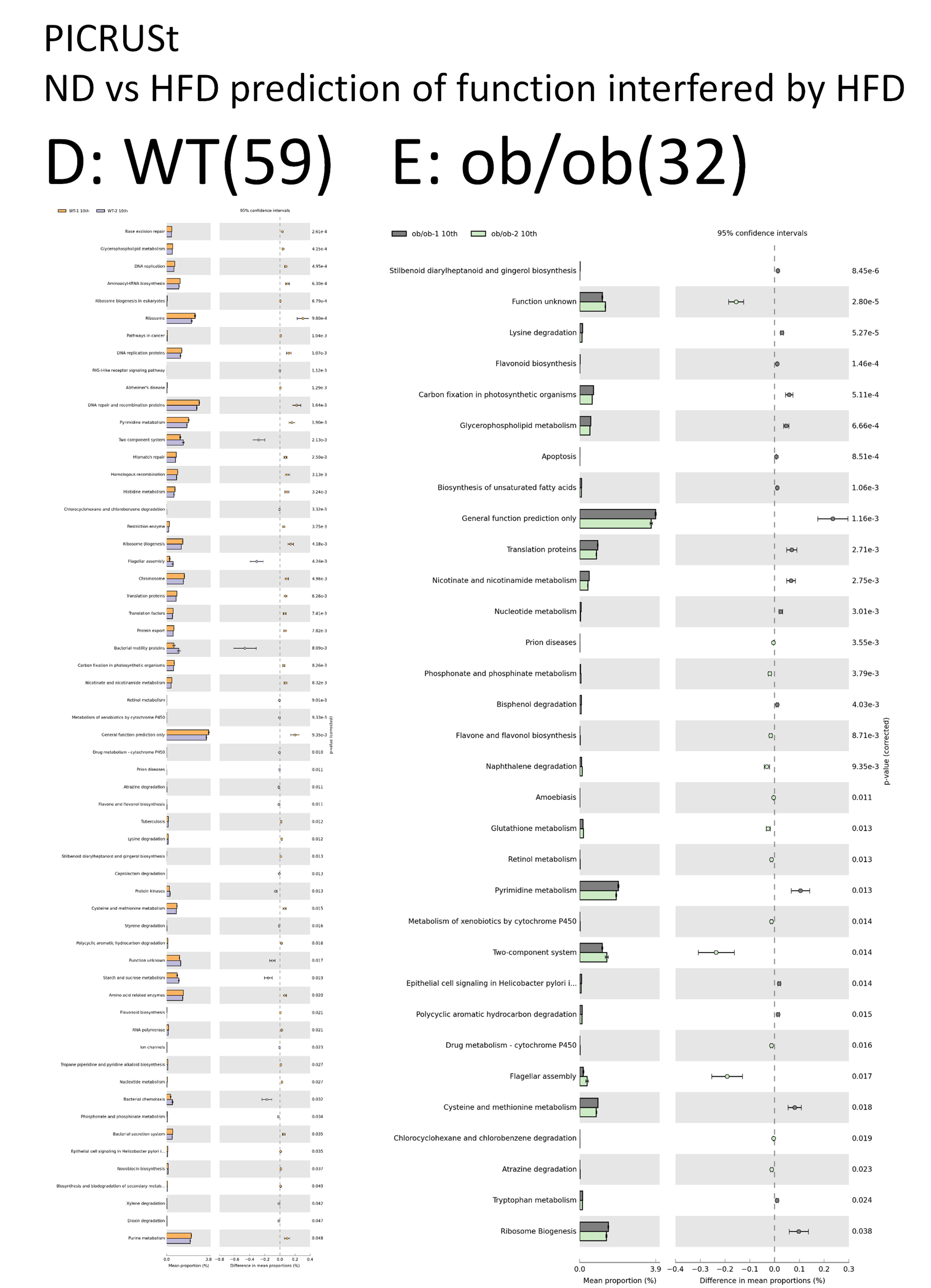


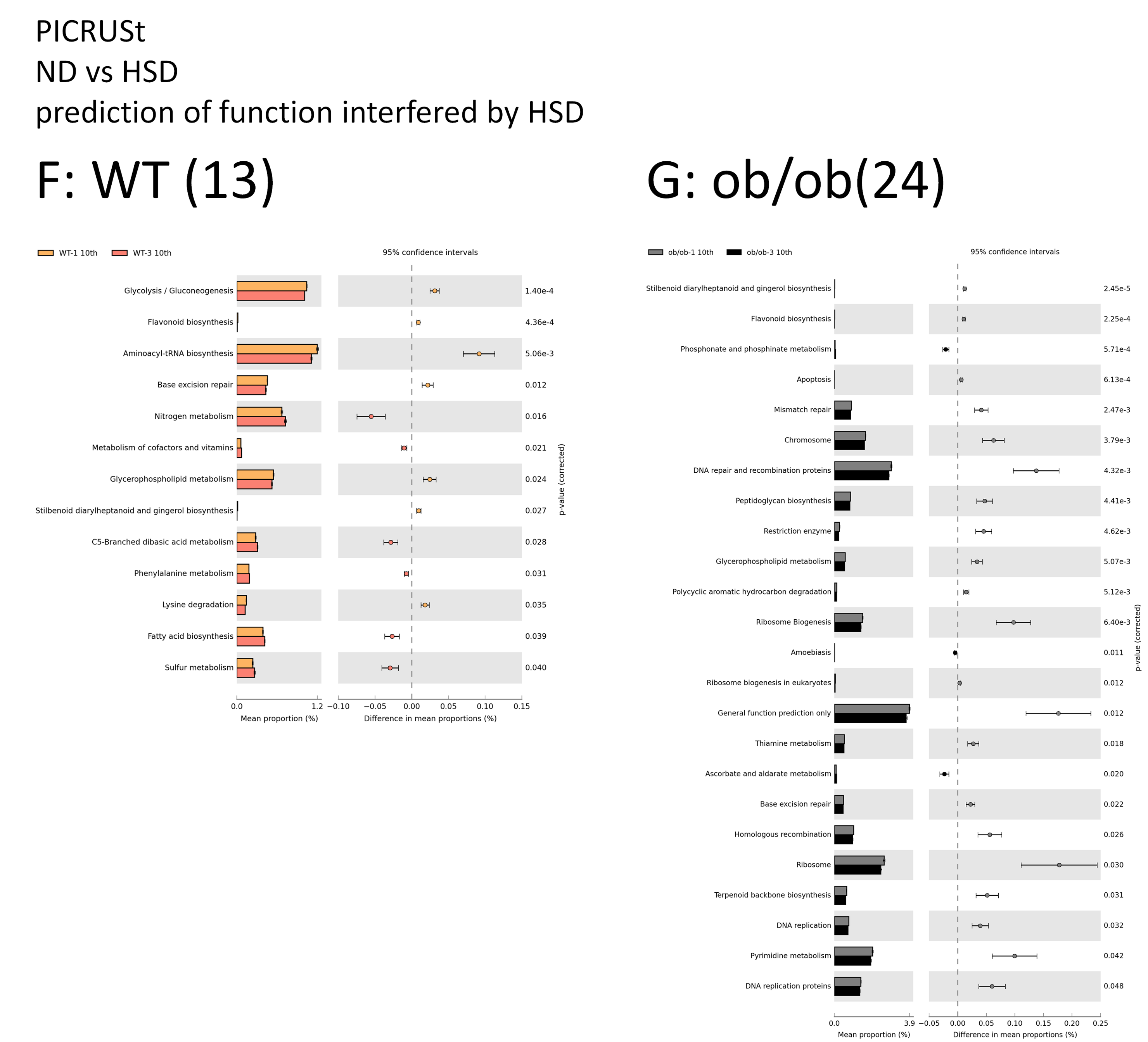


**Supplementary Figure S4 HUMAnN2 analysis for KEGG pathway prediction.**

All of the 16s rRNA gene sequencing data were predictably profiled by HUMAnN2 (The HMP Unified Metabolic Analysis Network 2) using the KEGG (Kyoto Encyclopedia of Genes and Genomes) pathway database and statistically analyzed and represented graphically with the STAMP v2.1.3 software (Dalhousie University, Halifax, Canada). (A) WT-ND vs ob/ob-ND (B) WT-HFD vs ob/ob-HFD (C) WT-HSD vs ob/ob-HSD (D) WT-ND vs WT-HFD (E) ob/ob-ND vs ob/ob-HFD (F) WT-ND vs WT-HSD (G) ob/ob-ND vs ob/ob-HSD.


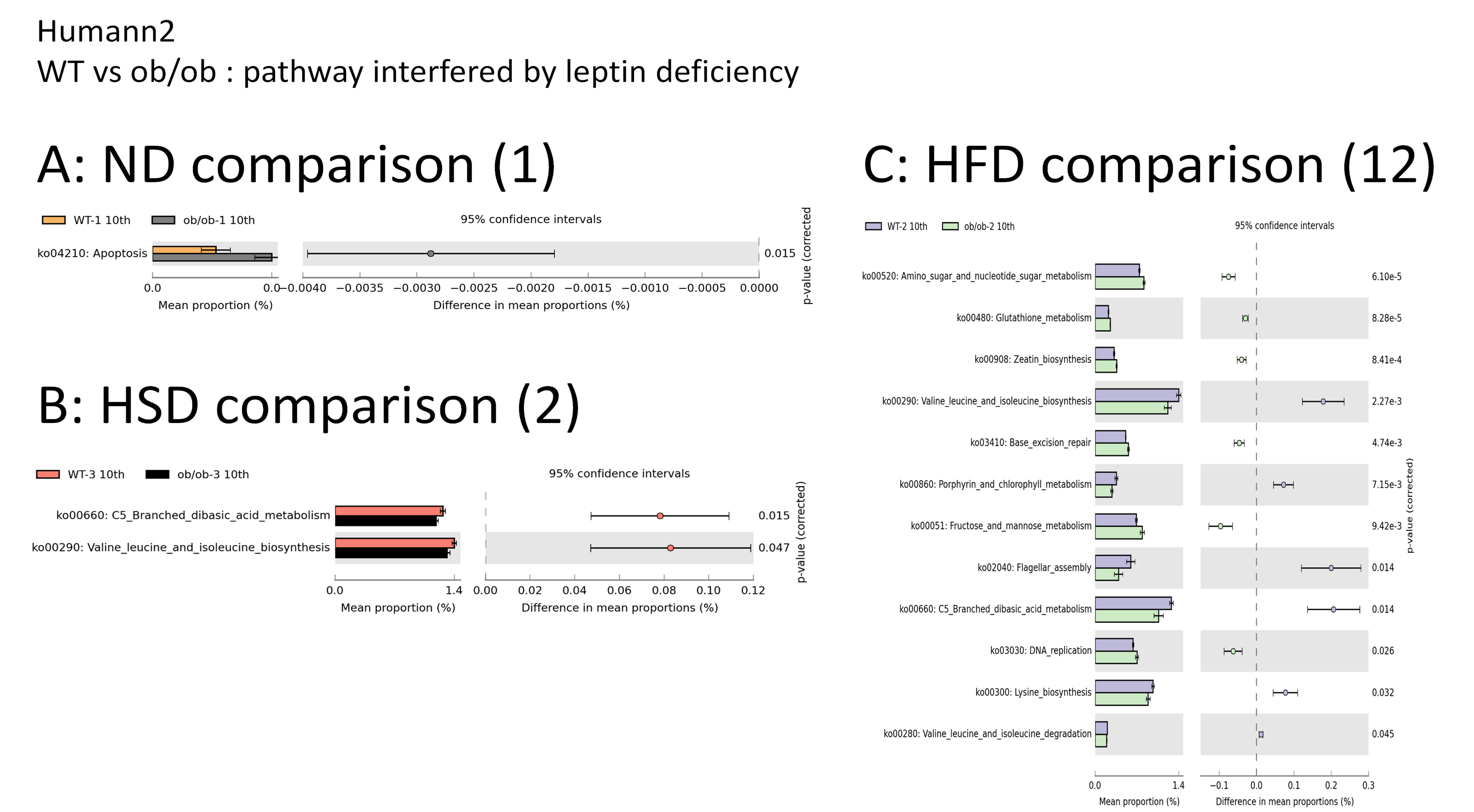


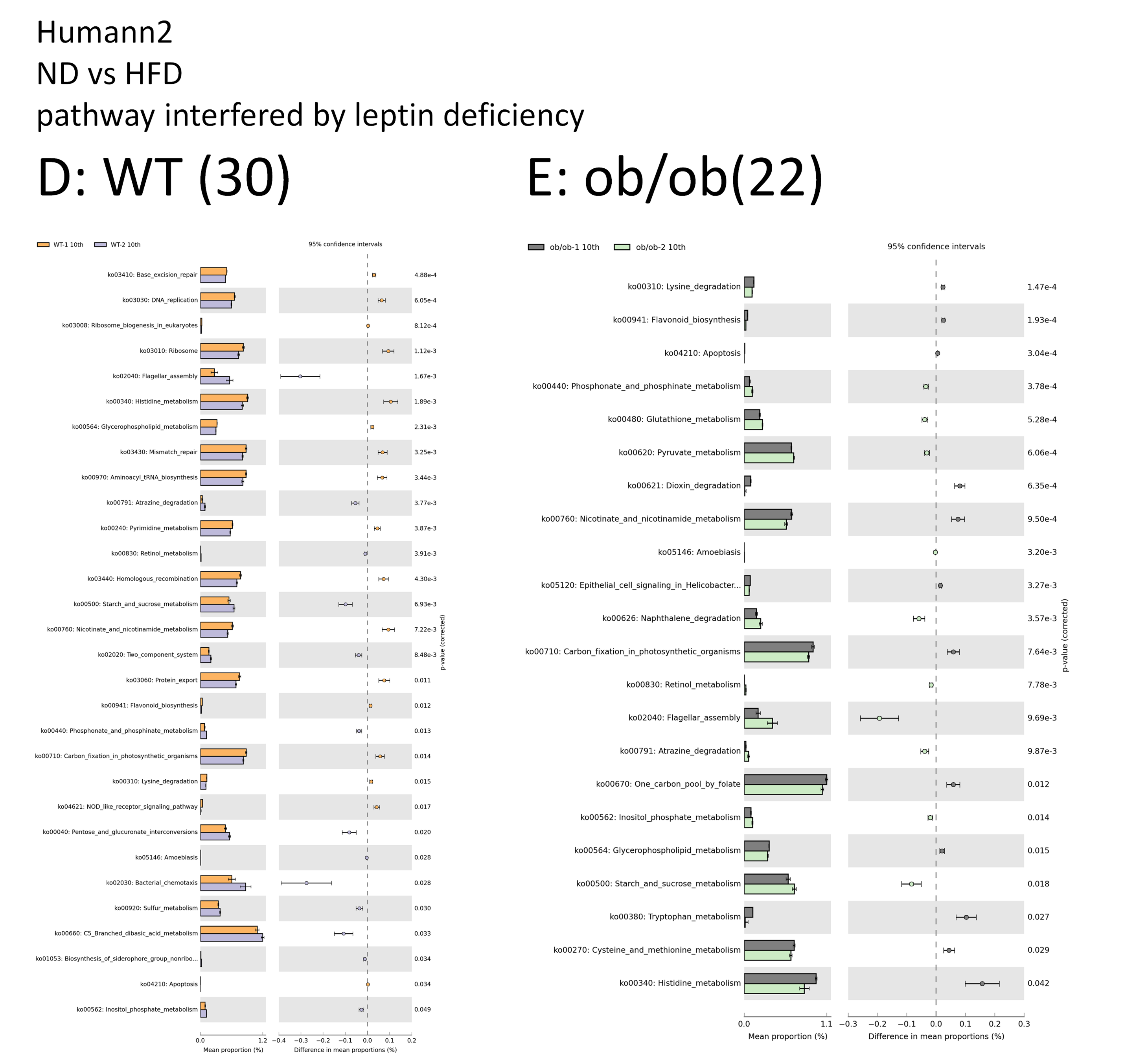


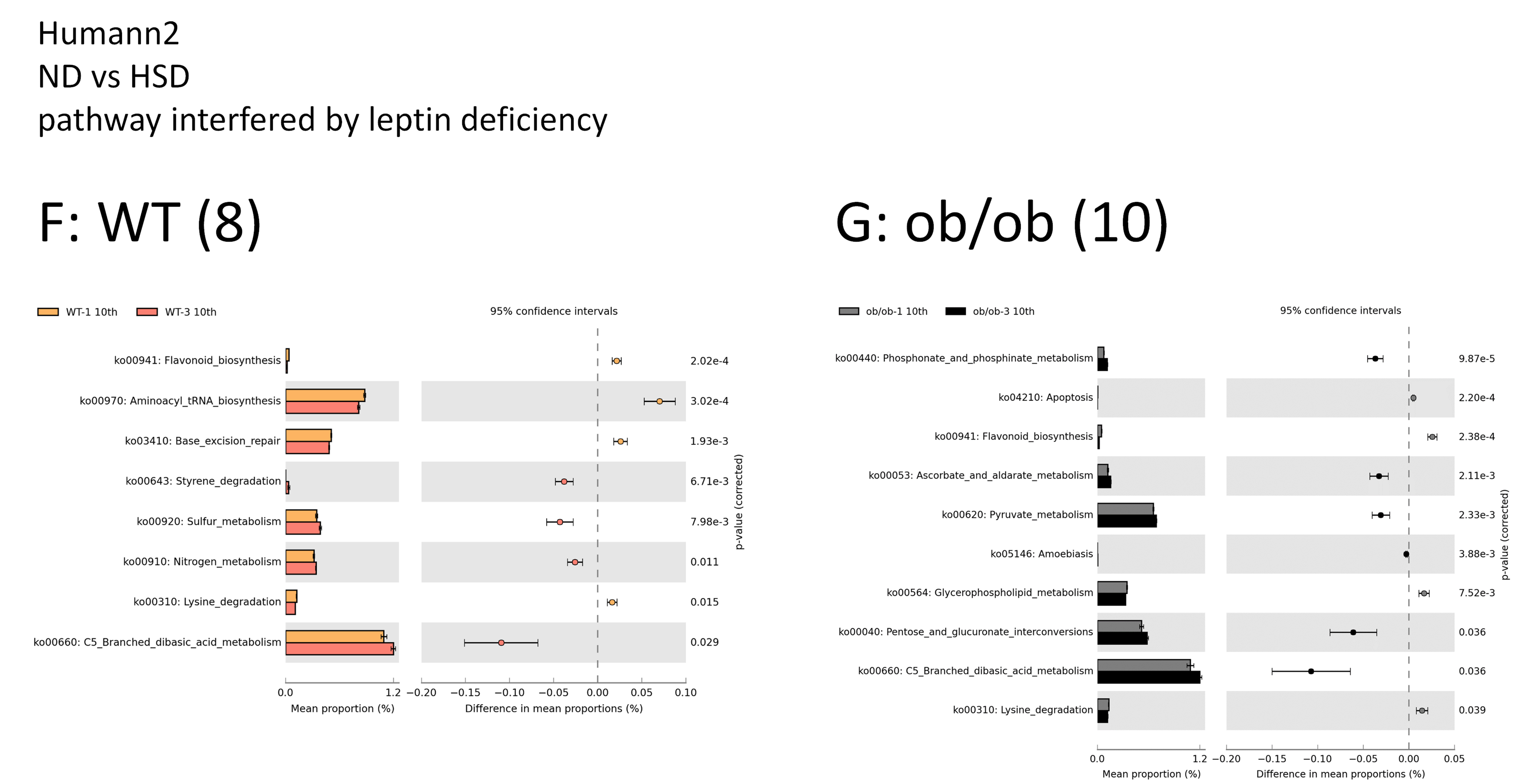

Supplement: Supplementary file 1 [file Table_1.DOCX]
